# Preserving low perfusion during surgical liver blood inflow control prevents hepatic microcirculatory dysfunction and irreversible hepatocyte injury in rats

**DOI:** 10.1038/srep14406

**Published:** 2015-09-24

**Authors:** Chong-Hui Li, Yong-Wei Chen, Yong-Liang Chen, Li-Bin Yao, Xin-Lan Ge, Ke Pan, Ai-Qun Zhang, Jia-Hong Dong

**Affiliations:** 1 Department and Institute of Hepatobiliary Surgery, Chinese PLA General Hospital, Chinese PLA Medical School, Beijing, China

## Abstract

Hepatic ischaemia/reperfusion (I/R) injury is of primary concern during liver surgery. We propose a new approach for preserving low liver blood perfusion during hepatectomy either by occlusion of the portal vein (OPV) while preserving hepatic artery flow or occlusion of the hepatic artery while limiting portal vein (LPV) flow to reduce I/R injury. The effects of this approach on liver I/R injury were investigated. Rats were randomly assigned into 4 groups: sham operation, occlusion of the portal triad (OPT), OPV and LPV. The 7-day survival rate was significantly improved in the OPV and LPV groups compared with the OPT group. Microcirculatory liver blood flow recovered rapidly after reperfusion in the OPV and LPV groups but decreased further in the OPT group. The OPV and LPV groups also showed much lower ALT and AST levels, Suzuki scores, inflammatory gene expression levels, and parenchymal necrosis compared with the OPT group. An imbalance between the expression of vasoconstriction and vasodilation genes was observed in the OPT group but not in the OPV or LPV group. Therefore, preserving low liver blood perfusion by either the OPV or LPV methods during liver surgery is very effective for preventing hepatic microcirculatory dysfunction and hepatocyte injury.

In liver surgery, Pringle maneuver, i.e., clamping of the portal triad, is often used to reduce intraoperative bleeding during hepatectomy. However, Pringle maneuver produces ischemic/reperfusion (I/R) injury to the remaining liver and intestinal congestion^1^,^2^. The temporary deprivation of blood flow to oxygen-dependent hepatocytes and nonparenchymal cells followed by reperfusion results in a complex series of events, including the release of reactive oxygen species, danger signals and inflammatory response cascades^3^,^4^. These events lead to intrahepatic microcirculatory disturbance, perfusion failure, hepatocyte death and ultimately organ dysfunction^2^,^5^. I/R injury directly affects patient outcomes and increases morbidity and mortality, particularly in patients with chronic liver diseases such as cirrhosis and cholestasis.

Many strategies in reducing liver I/R injury have been developed in experimental studies, such as ischaemic pre-conditioning and pharmacologic agents targeting microcirculation, oxidative stress, proteases, and inflammation^6^,^7^. However, it is rare that these approaches are successfully applied in clinical practice. The liver is unique in that it is supplied by two distinct blood inflow systems, the portal vein (PV), contributing 70–80%, and the hepatic artery (HA), contributing 20–30% of the total hepatic blood flow. Whereas portal venule ends directly in the sinusoid and becomes the primary feeder of hepatic sinusoids. Hepatic artery first supports the peribiliary plexus, which then drains into sinusoids. When the portal vein is clamped the sinusoidal blood flow decreased markedly. On the other hand, 80% of the hepatic sinusoidal flow is maintained after clamping the hepatic artery in the rat. Despite an overall reduction of liver blood flow upon PV flow restriction, HA is capable of meeting hepatic tissue oxygen demand, as indicated by maintained values of tissue PO_2_^8^,^9^.

We therefore proposed a method of preserving low liver blood perfusion by occluding the PV while preserving HA flow (the OPV method) to reduce liver I/R injury and control intraoperative blood loss. In our previous studies^9^,^10^, we have demonstrated that the OPV method significantly reduces hepatic I/R injury and improves regeneration of the remaining portion of the liver without an apparent increase in liver resection-related blood loss compared with the total blood inflow occlusion (Pringle maneuver) method. In this study, we also tested another method of preserving low liver blood perfusion, in which the HA is occluded and PV flow is limited (the LPV method) to reduce liver I/R injury, control intraoperative blood loss and avoid intestinal congestion. This method can also be used in cases in which HA flow is greater than normal, such as what occurs in cirrhotic livers.

The aim of the present study was to investigate the effects of preserving low liver blood perfusion, particularly using the LPV method, on microcirculatory LBF, sinusoidal endothelial function and histological injury in a rat liver I/R model.

## Materials and Methods

### Animals

Adult male Wistar rats weighing 230 to 270 g were obtained from the Experimental Animal Centre of the Academy of Military Medical Science (Beijing, China). All experimental procedures were carried out in accordance with the recommendations of the Guide for the Care and Use of Laboratory Animals of the National Institutes of Health and were approved by the Committee on Ethics of Animal Experiments of the Chinese PLA General Hospital.

### Experimental Groups and Sample Collection

The animals were randomly divided into the following four groups: sham operation (SO), occlusion of the portal triad (OPT), occlusion of PV with preservation of HA flow (OPV), and occlusion of HA with limitation of PV flow (LPV). For survival analysis, the liver blood inflow was occluded for 1 h, 1.5 h, 2 h, 2.5 h or 3 h. Survival was monitored for 7 days after surgery. For sample collection, the occlusion time was set for 2 h, and at 6 h, 24 h, 3 d and 7 d after reperfusion blood samples were collected from the inferior vena cava for liver enzyme analyses. Each ischaemic liver lobe was cut into two pieces, which were used for gene expression and histological assessments.

### Surgical Procedures

**Figure 1:**
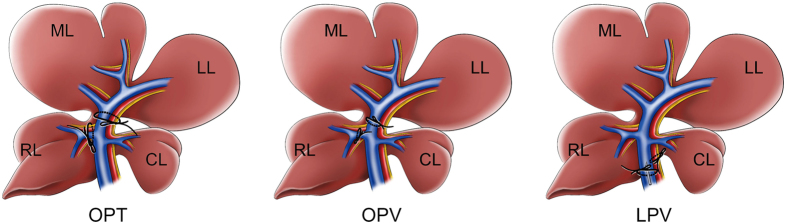
Graphical illustration of different liver vascular clamping methods used in rats. **OPT**, Occlusion of the portal triad (OPT) at the pedicles of the left lobe (LL), median lobe (RL) and right lobe (RL) with no occlusion of the caudate lobe (CL). **OPV**, Occlusion of the portal vein with preservation of hepatic artery flow (OPV). **LPV**, Occlusion of the hepatic artery with limitation of portal vein flow (LPV) in the common pedicles of the rat liver. This figure was drawn by Chong-Hui Li.

In the OPT and OPV groups, a rat model with I/R to 90% of the liver was used^11^. In this rat model, the caudal lobe, which accounts for approximately 10% of the liver, was not occluded because maintaining the caudal lobe as a passage for portal blood reduces intestinal congestion during liver ischaemia. After overnight fasting with free access to water, the rats were anaesthetized by continuous ether inhalation. Their body temperatures were maintained with a heating pad throughout the operation and monitoring periods. In the OPT group, the hepatic pedicle of the left and median lobes as well as that of the right lobe were gently isolated under a surgical microscope and temporally clamped with two lengths of “0” surgical suture tied in bowknots (Fig. 1A). In the OPV group, only the PV was isolated and clamped (Fig. 1B). In the LPV group, the PV in the common liver pedicle was surgically isolated and stenosed by tying a 3–0 silk suture to a 23-gauge needle, which was placed alongside the vein. The needle was then removed to generate a calibrated constriction of the PV^12^ (Fig. 1C). In the SO group, the pedicles were isolated but not clamped. Occlusion was verified visually by colour changes in the liver lobes. A simple continuous suture was used to close the peritoneal skin, and the rats were returned to their cages. After different durations of ischaemia, the peritoneal skin was reopened to untie the bowknots and initiate reperfusion. After the above procedures, the caudal lobe was resected to avoid its affect on the assessment of liver injury. For the LPV group, the PV flow was measured with a Transonic T402 flow metre before and after stenosis. There was an approximately 70–80% reduction in PV flow after stenosis.

### LBF Assessment with Laser Speckle Contrast Imaging (LSCI)

Microcirculatory LBF was assessed using a moorFLPI-2 Full-Field Laser Perfusion Imager (Moor Instruments, Axminster, UK) according to the methods used in our previous study^13^. LBF was measured at the following 4 time points: 5 min after laparotomy, which was considered the baseline value for LBF (Baseline); 5 min after the start of ischaemia (I-5 min); 5 min before the end of 120 min of ischaemia (I-115 min); and 5 min after the start of reperfusion (R-5 min). The manufacturer’s software (moorFLPI-2 Review V4.0, Moor Instruments, Axminster, UK) was used to analyse the images and quantify perfusion in an arbitrary laser speckle perfusion unit (LSPU).

### Blood Biochemical Analysis

Levels of serum alanine aminotransferase (ALT) and aspartate aminotransferase (AST) were measured using a Cobas 8000 serum analyser (Roche Diagnostics, Manheim, Germany).

### Histological Assessment

Liver specimens were embedded in paraffin, cut into 5 μm sections, and stained with haematoxylin and eosin. The histological severity of I/R injury was graded using a modification of Suzuki’s criteria^14^, as described in Table 1. The necrotic area was also quantitatively assessed using Image-Pro Plus v 6.0 (Media Cybernetics, Rockville, MD, USA). The results are presented as the percentage of necrotic area with respect to the entire area of the section. Immunostaining was performed for Ki-67 (BD Pharmingen, Franklin Lakes, NJ, USA) and cleaved caspase-3 (Cell Signaling Technology, Beverly, MA, USA) by staining with DAB followed by counter-staining with haematoxylin. The Ki-67 labelling index was determined as the percentage of Ki-67-positive hepatocytes per the total number of hepatocytes in 5 random visual fields (400×). Immunofluorescence was performed for rat endothelial cell antigen 1 (RECA-1) (Serotec, Oxford, UK).

### Indocyanine Green Plasma Disappearance Rate (ICG-PDR)

ICG (Dandong Medical Co. Ltd., Liaoning, China) was dissolved in sterile water to a final concentration of 2.5 mg/mL. The inferior caval vein was used for blood sample collection performed at 1, 3, and 5 min after freshly prepared ICG was injected into the femoral vein (2.5 mg/kg). After centrifugation, the plasma samples were diluted and measured spectrophotometrically at 805 nm (Beckman Coulter DU640, Brea, CA, USA). The PDR was derived from the slope of a semi-logarithmic decay curve^15^.

### Quantitative Real-Time PCR

Total RNA was extracted from the frozen liver tissues using an RNASimple Total RNA Kit (Tiangen Biotech, Beijing, China). Four micrograms of RNA were reverse-transcribed with a RevertAid First Strand cDNA Synthesis Kit (Fermentas International Inc., Burlington, Canada). Quantitative real-time PCR amplification was performed with an SYBR Premix Ex Taq II (TaKaRa Bio Inc, Dalian, China) using a StepOnePlus Real-Time PCR System (Applied Biosystems, CA, USA). The primers used are shown in Supplementary Table S1 online. The relative gene expression levels were normalized to the expression of GAPDH and calculated using the 2-ΔΔCt method as previously described^16^. The results are expressed as fold changes versus the SO group.

### Western Blot Analysis

Frozen liver tissues were homogenized in RIPA buffer (Cell Signaling Technology, Beverly, MA, USA) mixed with phenylmethanesulfonyl fluoride, proteinase and phosphatase inhibitor cocktails (Roche, Basel, Switzerland). Protein extracts (40–100 μg) were separated in 8%–12% SDS-PAGE gels and transferred to nitrocellulose membranes. The blots were subsequently blocked for 1 h and probed overnight at 4 °C with antibodies recognizing endothelin-1 (ET-1) (Abcam, Hong Kong, China), ICAM-1 (Santa Cruz Biotechnology, Santa Cruz, CA, USA), endothelial nitric oxide synthase (eNOS) (Cell Signaling Technology, Beverly, MA, USA) and phosphorylated eNOS (p-eNOS) at Ser1176 (BD Transduction Laboratories, Lexington, KY, USA). Probing was followed by incubation with the corresponding horseradish peroxidase (HRP)-conjugated secondary antibodies (1:2000) for 1 h at room temperature. The blots were visualized by enhanced chemiluminescence. Protein expression was determined by densitometric analysis using a Gel-Pro Analyzer. The blots were assayed for the GAPDH (Sungene, Beijing, China) content as a standardization of sample loading. The degree of eNOS phosphorylation at Ser1176 was calculated as the ratio of the densitometry readings of p-eNOS to that of eNOS.

### Statistical Analysis

All results are expressed as the mean ± standard deviation. More than 2 groups were compared by one-way ANOVA with post hoc tests for multiple comparisons using SPSS version 17.0 (SPSS Inc., Chicago, IL, USA). When the criteria for parametric testing were violated, the appropriate nonparametric test (Mann-Whitney U-test) was used. For the survival experiment, the log-rank test was conducted. Values of P < 0.05 were considered to be significant.

## Results

### Preserving Low Perfusion Significantly Extended the Ischaemic Tolerance Time of Rat Liver

**Figure 2:**
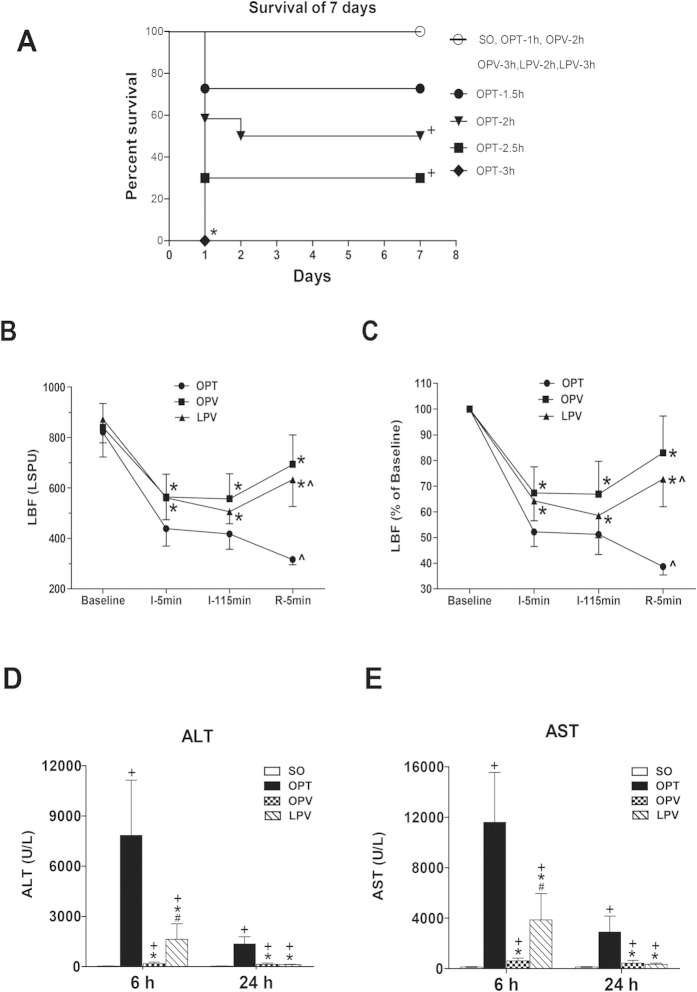
The survival rate and changes in microcirculatory liver blood flow and serum aminotransferase levels detected with the use of different liver vascular clamping methods in rats. (**A**) Survivorship curves, showing the survival rates of rats in the SO, OPT, OPV and LPV groups exposed to different vascular clamping times of up to 7 days post-operation (n = 8–12 in each subgroup). **+** vs. the SO group, P < 0.05. (**B**,**C**) Alterations in microcirculatory liver blood flow (LBF) in the SO, OPT, OPV and LPV groups, as measured with laser speckle contrast imaging at 5 min after laparotomy (Baseline), at 5 min after the start of ischaemia (I-5 min), at 5 min before the end of ischaemia (I-115 min) and at 5 min after the start of reperfusion (R-5 min). LBF is expressed as a raw arbitrary perfusion unit (LSPU) in B and as a percentage of the baseline level in C. n = 8 for each group. * vs. OPT, P < 0.05; ^ vs. Baseline, P < 0.05. (**D**,**E**) Serum alanine aminotransferase (ALT) (**D**) and aspartate aminotransferase (AST) levels (**E**) in the rats from the SO, OPT, OPV and LPV groups subjected to 2 h of the different liver blood inflow occlusion and 6 h and 24 h of reperfusion. n = 6 for each group. **+** vs. SO, P < 0.05; * vs. OPT, P < 0.05; # vs. OPV, P < 0.05.

It is possible to occlude the total blood inflow to the other liver lobes for at least several hours With the caudal lobe of the rat liver unclamped as a bypass for portal blood. In the OPT group, occlusion of both PV and HA flow for 1 h, 1.5 h, 2 h, 2.5 h and 3 h resulted in 7-day survival rates of 100%, 73%, 50%, 30% and 0%, respectively (OPT-2 h, OPT-2.5 h and OPT-3 h vs. SO; P < 0.05) (Fig. 2A). However, in the OPV and LPV groups, the 7-day survival rates corresponding with 2 h and 3 h of occlusion were all 100%. These results indicated that preserving low blood perfusion to the liver significantly extended the ischaemic tolerance time of the rat liver and contributed to the survival advantages of the OPV and LPV groups. Considering that the clinical liver resection time is usually less than 2 h and that with 2 h of occlusion the OPT group experienced a moderate degree of I/R injury, 2 h of occlusion time was used in the subsequent experiments.

### Microcirculatory LBF Changes Measured with LSCI

There were no significant differences among the baseline LBFs of the OPT, OPV and LPV groups (P > 0.05) (Fig. 2B). The LBFs decreased substantially at 5 min post-occlusion. The LBF of the OPT group was significantly lower than those of the OPV and LPV groups (P < 0.05). During the 2 h of different extents of ischaemia, the LBFs of the OPT and OPV groups exhibited no obvious changes, and that of the LPV group decreased slightly but not significantly. Following the start of reperfusion, the LBF of the OPT group decreased further, but those of the OPV and LPV groups increased and were slightly higher in the OPV group compared with the LPV group. These results demonstrated the rapid recovery of microcirculatory perfusion. Similar changes in LBF were observed when the raw arbitrary LBF value of each rat liver was expressed as a percentage of its baseline value (Fig. 2C).

### Liver Damage Following the Use of Different Liver Blood Inflow Occlusion Methods

At 6 h post-reperfusion, the OPT group had the highest ALT and AST levels among the four experimental groups, which were several times higher than the levels detected in the OPV and LPV groups (Fig. 2D,E). The ALT and AST levels of the OPV group were slightly higher than those of the SO group but were much lower than those of the LPV group. At 24 h after reperfusion, the OPT group still showed higher ALT and AST levels than the other groups, and there were no differences between the OPV and LPV groups. These results demonstrated that preserving low blood perfusion was very effective in reducing liver I/R injury compared with total liver blood occlusion, and that a lack of HA perfusion caused more damage than a lack of PV flow, as reflected by the release of more enzymes in the LPV group compared with the OPV group.

### Histological Changes in the Rat Liver Following the Use of Different Blood Inflow Control Methods

**Figure 3:**
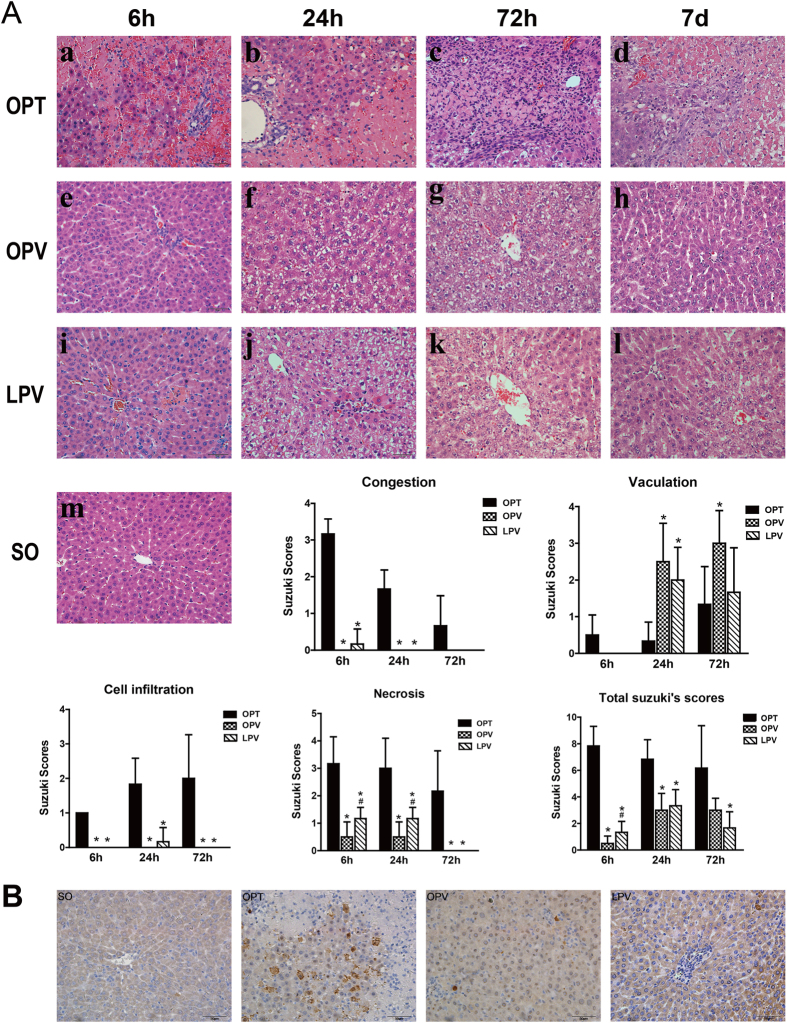
Histopathological analysis of the rat livers after the use of different liver vascular clamping methods. All rats from the SO, OPT, OPV and LPV groups were subjected to 2 h of different liver blood inflow occlusion methods and 6 h, 24 h, 72 h and 7 d of reperfusion. n = 6 for each subgroup. (**A**) Representative liver sections stained with haematoxylin and eosin (400×) and their Suzuki’s scores. Note the presence of hepatocellular necrosis, as evidenced by pyknotic nuclei, cytoplasmic blanching, the loss of distinct hepatocellular borders in (a–d) and hepatocellular vacuolization in (f,g,j,k). For Suzuki scoring: * vs. OPT, # vs. OPV, P < 0.05. (**B**) Hepatocellular apoptosis was assessed by immunohistochemical staining of cleaved caspase-3.

Histological sections from the SO rats and the rats subjected to 2 h of no-flow or low-flow liver ischaemia were evaluated for I/R injury according to Suzuki’s criteria (Fig. 3A). In contrast with the normal lobule structure of the SO group, the most prominent morphological changes in the OPT group were sinusoidal congestion and hepatocellular necrosis. At 24 h of reperfusion, the average necrotic area was 7.64 ± 9.22% following 1.5 h of ischaemia and 26.4 ± 12.5% following 2 h of ischaemia. However, in the OPV and LPV groups, the lobular structure remained intact and there was no obvious sinusoidal congestion. Necrotic hepatocytes were rare in the OPV group and were occasionally observed as scattered foci in the LPV group. Significant hepatocellular vacuolization was observed in the OPV and LPV groups at 24 h and 3 d of reperfusion. The total Suzuki score of the OPT group was also significantly higher than those of the OPV and LPV groups at 6 h, 24 h and 3 d post-reperfusion (Fig. 3A).

In addition, hepatocellular apoptosis was assessed by immunostaining of cleaved caspase-3 (Fig. 3B). Numerous apoptotic hepatocytes were observed in the OPT group, mainly around the necrotic hepatocytes. In contrast, apoptotic hepatocytes were infrequently observed in the OPV and LPV groups. These results suggested that total liver blood inflow occlusion resulted in severe irreversible hepatocellular damage characterized by necrosis or apoptosis.

### Liver Function and Regeneration Following the Use of Different Blood Inflow Control Methods

**Figure 4:**
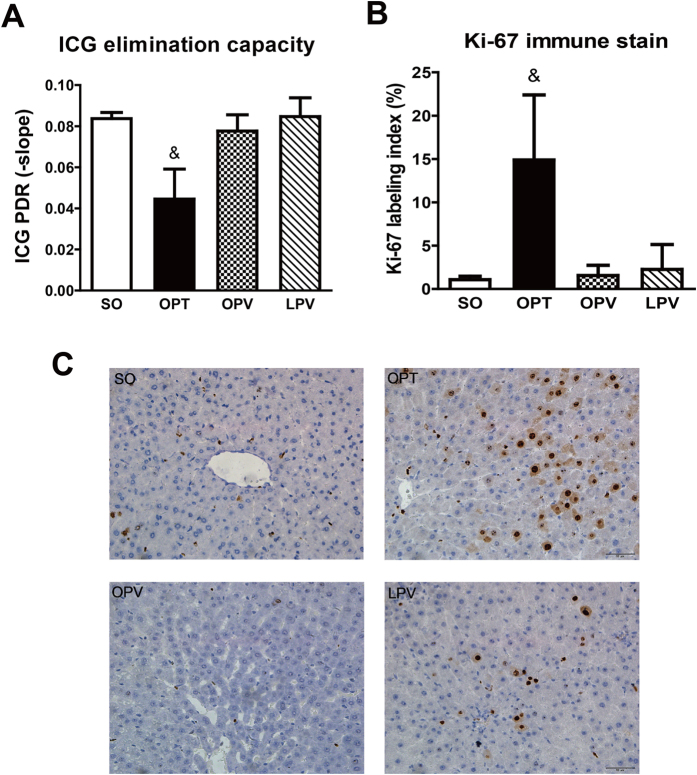
Assessments of liver function and regeneration. The indocyanine green plasma disappearance rate (ICG-PDR) (**A**) and immunohistochemical staining for Ki-67 (**B,C**) in the rat livers of the rats in the SO, OPT, OPV and LPV groups subjected to 2 h of the different liver blood inflow occlusion methods and 24 h of reperfusion. n = 6 for each group. & vs. SO, OPV and LPV group, P < 0.05. Nuclei with deposition of brownish pigment were considered to be labelled for Ki-67 (**C**) (400×).

The ICG-PDR was assessed as a marker of the maximum elimination capacity of the liver. As illustrated in Fig. 4A, the ICG-PDRs of the SO, OPV and LPV groups were not significantly different, but that of the OPT group was much lower than those of the other three groups at 24 h after reperfusion (P < 0.05), suggesting the presence of minimal damage to the elimination capacities of the hepatocytes in the OPV and LPV groups.

A liver regenerative response was detected by Ki-67 immunostaining (Fig. 4B,C). The Ki-67 labelling index was significantly higher in the OPT group than those in the OPV and LPV groups at 24 h and on day 3 post-operation (P < 0.05), whereas Ki-67-positive hepatocytes in the SO, OPV and LPV groups were minimal (P > 0.05). These results suggested that liver damage-induced regeneration of hepatocytes occurred in the OPT group.

### Analysis of the Gene Expression in the Rat Liver Following the Use of Different Blood Inflow Control Methods

**Figure 5:**
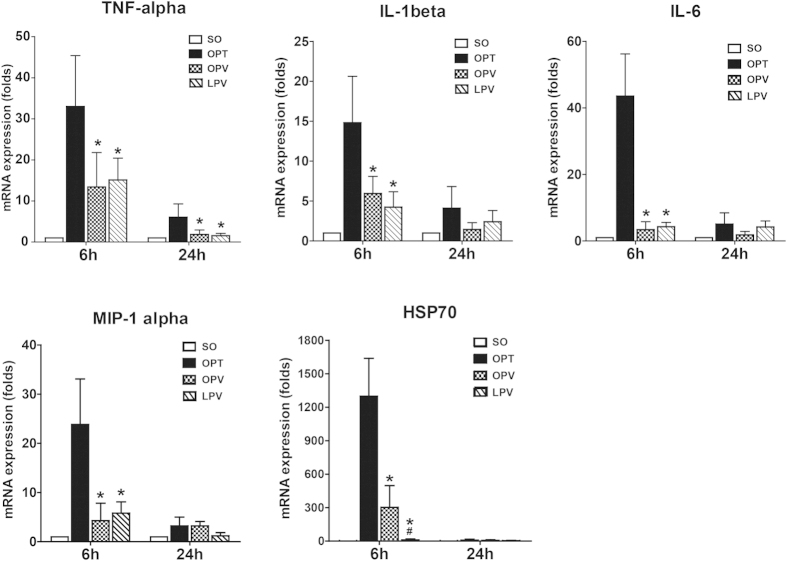
Assessment of inflammatory cytokine gene expression. TNF-α, IL-1β and IL-6, chemokine MIP-1α and heat shock protein 70 (HSP70) gene expression levels in livers of the rats from the SO, OPT, OPV and LPV groups were detected by qRT-PCR. Values were normalized to GAPDH expression and are expressed as the mean fold change ± SD versus the SO group. n = 6 for each group. * vs. OPT group, P<0.05; # vs. OPV group, P < 0.05.

The liver expression levels of several genes related to I/R injury, including the inflammatory cytokines TNF-α, IL-1β and IL-6, chemokine macrophage inflammatory protein-1 α (MIP-1α), and heat shock protein 70 (HSP70), were assessed by qRT-PCR. As shown in Fig. 5, substantially increased gene expression levels of TNF-α, IL-1β, IL-6, MIP-1α and HSP70 were observed in the OPT group at 6 h post-operation. The mRNA levels of these genes were also increased in the OPV and LPV groups, but they were expressed at much lower levels than those in the OPT group (P < 0.05). Comparison of the expression of these genes between the OPV and LPV groups revealed that only HSP70 in the OPV group was expressed at a significantly higher level compared with that in the LPV group (P < 0.05), suggesting that the stress response in the OPV group was more severe than that in the LPV group.

### Assessment of Endothelial Gene Expression in the Rat Liver Following the Use of Different Blood Inflow Control Methods

**Figure 6:**
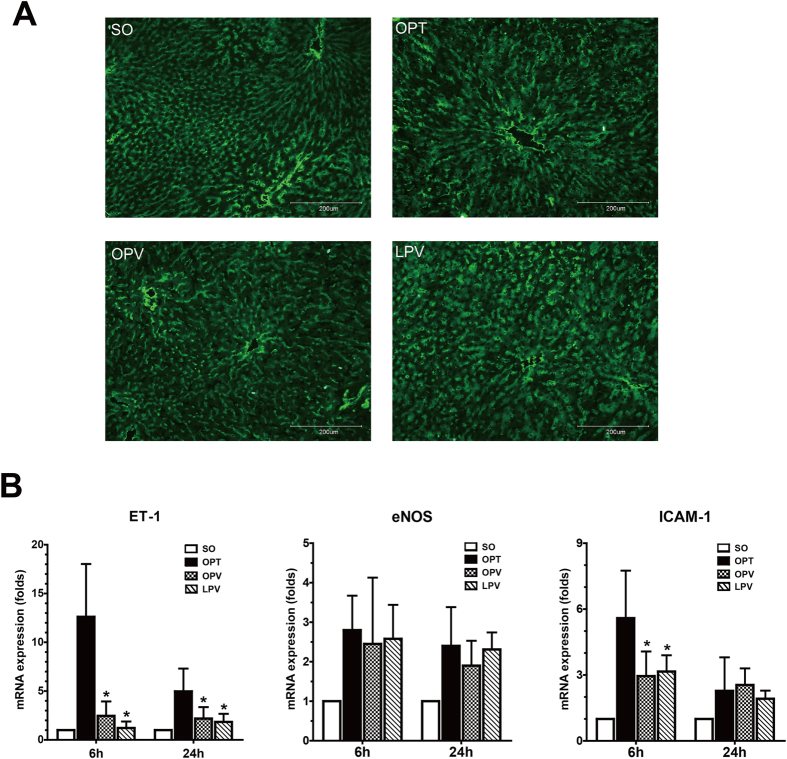
Detection of the changes in liver sinusoidal endothelum by immunofluorescence staining and qRT-PCR. (**A**) Immunofluorescence staining of the rat liver endothelial marker RECA-1 (200×). (**B**) Detection of expression of the endothelial function-related genes ET-1, ICAM-1 and eNOS by qRT-PCR in the livers of the rats in the SO, OPT, OPV and LPV groups at 6 h and 24 h after reperfusion. Values were normalized to GAPDH expression and are presented as the mean fold change ± SD versus the SO group. n = 6 for each group. * vs. OPT group, P < 0.05.

**Figure 7:**
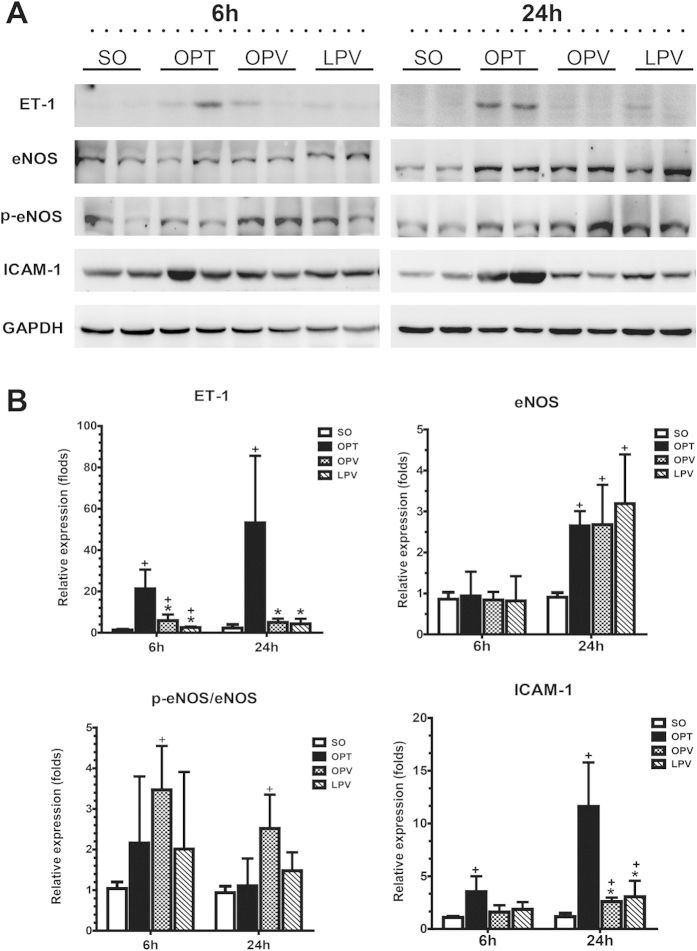
Detection of changes in liver sinusoidal endothelum by Western blot analysis. The endothelial function-related genes ET-1, ICAM-1, eNOS and eNOS phosphorylation (p-eNOS) in the livers of the rats in the SO, OPT, OPV and LPV groups at 6 h and 24 h after reperfusion were assessed by Western blotting. The relative protein expression levels were normalized to GAPDH expression and are presented as the mean fold change ± SD versus the SO group. n = 6 for each group. **+** vs. SO group, P < 0.05; * vs. OPT group, P < 0.05.

To further characterize the effect of preserving low perfusion on intrahepatic microcirculation, we first examined whether there was a loss in sinusoidal endothelial cells by immunofluorescence staining of the endothelial marker RECA-1. However, we did not find significant differences among the four experimental groups in RECA-1 expression (Fig. 6A), indicating that no significant sinusoidal endothelial cell loss occurred. We then assessed the gene and protein expression of ET-1, eNOS and ICAM-1 and eNOS phosphorylation in the reperfused liver samples. We found that ET-1 gene and protein expression were both markedly upregulated in the OPT group but only slightly upregulated in the OPV and LPV groups (Fig. 6B and Fig. 7A,B). Parallel changes were observed for the gene and protein expression of eNOS in the OPT, OPV and LPV groups. Furthermore, p-eNOS, the active form of this protein, was slightly increased only in the OPV group. However, the gene and protein expression levels of ICAM-1 were increased to much higher levels in the OPT group than in the OPV and LPV groups. Overall, the liver endothelium of the OPT group exhibited significantly upregulated expression of the endothelial vasoconstrictor gene ET-1 and injury-related gene ICAM-1 compared with their expression in the OPV and LPV groups.

## Discussion

In the present study, a surgical selective liver blood inflow control method was investigated for reducing liver-resection-related I/R injury. Induction of either reversible ischaemic injury or irreversible necrosis and apoptosis depends on the degree of ischaemia or the adequacy of oxygen supply to the liver^17^. To obtain a balance between oxygen supply and control of blood loss during hepatectomy, we proposed the strategy of maintaining low blood perfusion to the liver during this procedure. In the present study, we provide direct evidence that preserving low perfusion to the liver via occlusion of the PV while preserving HA flow or occlusion of the HA while limiting the PV is very effective in preventing I/R-induced hepatic microcirculatory dysfunction and irreversible hepatocellular injury.

Total PV occlusion induces intestinal congestion in rats and is lethal when the occlusion time exceeds 30 min^18^. The animal models that have been designed for avoiding portal congestion, such as the port-cava or portal-systemic intubation bypass models, are complicated^19^. In our rat liver I/R model, the caudate lobe was maintained as a passage for portal blood and was resected after reperfusion to prevent it from affecting the evaluation of liver function and injury. This model enables researchers to sustain ischaemia for a much longer period of time in up to 90% of the rat liver. As demonstrated in the present study, all rats successfully endured 1 h of no-flow ischaemia but failed to endure 3 h of no-flow ischaemia. In contrast, the 7-day survival rates in both the OPV and LPV groups were 100%, even for 3 h of low-flow ischaemia. These results demonstrated that supplying some oxygen to rat liver by preserving low perfusion significantly extended the safe ischaemic tolerance time.

I/R-induced impairment of hepatic microcirculation following reperfusion has been reported to be an important determinant of the occurrence of I/R injury^1^,^5^. LSCI is a recently marketed technique based on speckle contrast analysis^20^ that provides non-contact full-field imaging over wide areas with excellent spatial and temporal resolutions^13^,^21^,^22^. In the present study, alterations in microcirculatory LBF caused by the use of different vascular clamping methods in the liver were monitored using LSCI, confirming that low blood perfusion was preserved during ischaemia in the OPV and LPV groups. Following reperfusion, the LBF of the OPT group did not increase; rather, it decreased further, indicating microcirculatory dysfunction. In contrast, the LBFs of the OPV and LPV groups increased markedly following reperfusion, suggesting the rapid recovery of microcirculatory LBF. The observed reduction in sinusoidal perfusion in the OPT rats may have been due to the obstruction of sinusoids by entrapped blood cells or thrombotic material in addition to the swelling of perisinusoidal cells^23^. These results demonstrate that maintaining low liver perfusion for surgical liver blood control helped to preserve liver microcirculation function.

When total liver blood inflow is occluded, prolonged ischaemia results in severe damage and even necrosis of liver parenchyma. We found that the serum ALT and AST levels and Suzuki score were greatly increased in the OPT group compared with those in the OPV and LPV groups. Consistent with the presence of liver damage, liver function, as assessed by the ICG elimination capacity, was significantly reduced in the OPT rats compared with the OPV and LPV rats. In addition to induction of direct hepatocellular damage by ATP depletion, the obvious disturbance in microvascular perfusion in the OPT group was an important contributing factor to the large area of hepatocellular necrosis. This irreversible injury can be aggravated by a cascade of cellular and inflammatory events that eventually lead to organ dysfunction and mortality^24^. In contrast, in the OPV and LPV groups, preserving low perfusion only resulted in slight liver injury, mainly hepatocellular vacuolation, which recovered at several days post-operation. Hepatocyte regeneration was observed in the OPT group at 24 h post-operation and was likely a compensatory mechanism for the loss of necrotic hepatocytes. However, there was minimal hepatocyte regeneration in the OPV group and only occasional regeneration in the LPV group. Collectively, preserving low perfusion in the liver resulted in reversible liver injury.

ET-1 is a potent vasoconstrictor of microcirculation that is mainly produced by sinusoidal endothelial cells in the liver. The constricting action of endothelin balances with the dilating action of NO, which is produced constitutively by eNOS in the intact liver^25^,^26^. An elevated plasma ET-1 level has been observed after hepatic I/R and has been strongly correlated with events that aggravate reperfusion injury, such as sinusoidal constriction, a reduction in perfusion, and leukocyte–endothelium interactions^26^. Consistent with these previous findings, both the gene and the protein expression levels of ET-1 were found to be significantly elevated in the OPT group in our study. However, there was no significant increase in ET-1 expression in the OPV or LPV groups. The mRNA and protein expression levels of eNOS were only slightly upregulated in the OPT, OPV and LPV groups compared with those in the SO group. Only a slight increase in the eNOS phosphorylation was found in the OPV group. These alterations resulted in an obvious imbalance between the levels of ET-1 and eNOS-produced NO in the OPT group but not in the OPV or LPV groups. In addition, the ICAM-1 expression was much higher in the OPT group than that in the OPV and LPV groups. The elevations in ET-1 and ICAM-1 expression that occurred in the OPT group may have caused vasoconstriction and enhanced leukocyte- and platelet-endothelium interactions in the liver, aggravating hepatic microcirculatory disturbances following reperfusion. In contrast with the OPT group, the balanced expression of a microvascular regulatory gene in the OPV and LPV groups may have contributed to maintenance of microcirculatory function and avoidance of I/R injury.

A comparison of the OPV and LPV methods revealed minor differences. In the OPV group, low perfusion was maintained by HA blood, which is rich in oxygen, and the OPV group rat livers showed no significant hepatocyte necrosis or reduction in the ALT and AST level, and they exhibited higher eNOS phosphorylation than that in the LPV group rat livers. However, from a clinical perspective, the selection of the OPV or LPV method during hepatectomy may depend on the situation of the patient and liver disease. HA blood flow has been reported to be increased in patients with HBV-related liver cirrhotic or portal hypertension^27^. Increased blood loss may occur when the OPV method is used for liver resection in these patients; thus, the LPV method may be a better choice. For patients with poor liver function or severe liver injury because of hepatolithiasis or hilar tumours who are undergoing hepatectomy, OPV may be a better choice than LPV because it allows for increased hepatocyte protection. In addition, the LPV method may prevent accumulation of toxic substrates in the portal blood, thereby reducing I/R injury compared with the OPV method and Pringle maneuver.

When the OPV or LPV method is applied in liver resection, the porta hepatis must be dissected for HA or PV occlusion in contrast with the Pringle maneuver, adding complexity to these methods. For patients with blood vessel variation in the ligamentum hepatoduodenale or with abdominal adhesions due to previous surgery, dissection may increase surgical risks. Therefore, careful preoperative examination is needed in cases in which the OPV or LPV method is used.

Our surgical low liver perfusion rat model allowed for the detailed study of liver injury, haemodynamic alterations and the short-term molecular mechanisms involved in low-flow perfusion. The results demonstrate that occlusion of the PV with preservation of HA flow or occlusion of the HA with limitation of PV flow are two feasible methods to control for liver blood loss during liver resection. These methods strongly protect hepatic microcirculation and prevent irreversible hepatocyte injury. Therefore, from a surgical point of view, preserving low liver perfusion is easily achieved in liver resection, especially for patients with abnormal liver parenchyma, as an alternative to the Pringle maneuver or intermittent Pringle maneuver for liver blood loss control. Meanwhile the Pringle maneuver can also be easily adopted in cases in which stricter bleeding control is needed. These two methods are currently under clinical evaluation at our centre.

## Additional Information

**How to cite this article**: Li, C.H. *et al*. Preserving low perfusion during surgical liver blood inflow control prevents hepatic microcirculatory dysfunction and irreversible hepatocyte injury in rats. *Sci. Rep*. **5**, 14406; doi: 10.1038/srep14406 (2015).

## Supplementary Material

Supplementary Information

## Figures and Tables

**Table 1:** Modified Suzuki’s criteria.

Numerical assessment	Congestion	Vacuolation	Cell Infiltration	Necrosis
0	None	None	None	None
1	Minimal	Minimal	Minimal	Single cell
2	Mild	Mild	Mild	<30%
3	Moderate	Moderate	Moderate	<60%
4	Severe	Severe	Severe	>60%
