# Supplementary material for: Preserving low perfusion during surgical liver blood inflow control prevents hepatic microcirculatory dysfunction and irreversible hepatocyte injury in rats
Source: Sci Rep. 2015 Sep 24;5:14406. doi: 10.1038/srep14406 (PMC4585878; doi:10.1038/srep14406)
Supplement: Supplementary Information [file srep14406-s1.doc]

**Preserving low perfusion during surgical liver blood inflow control prevents hepatic microcirculatory dysfunction and irreversible hepatocyte injury in rats**

Chong-Hui Li, Yong-Wei Chen, Yong-Liang Chen, Li-Bin Yao, Xin-Lan Ge, Ke Pan, Ai-Qun Zhang, Jia-Hong Dong*

**Supplementary Materials**

**Table S1 Primer sequences used for gene expression analysis by qRT–PCR**

| **Genes** | **Forward(5′- 3′)** | **Reverse(5′- 3′)** |
| --- | --- | --- |
| TNF-α | AAATGGGCTCCCTCTCATCAGTTC | TCTGCTTGGTGGTTTGCTACGAC |
| IL-1 β | CACCTCTCAAGCAGAGCACAG | GGGTTCCATGGTGAAGTCAAC |
| IL-6 | ACAGCGATGATGCACTGTCAG | ATGGTCTTGGTCCTTAGCCAC |
| MIP-1α | GCGCTCTGGAACGAAGTCT | GAATTTGCCGTCCATAGGAG |
| HSP70 | GGCTAGAGACAGACTCTTGATGG | CTCAGTTTGTAGGGATGCAAGG |
| ET-1 | CATCTGGGTCAACACTCCCG | GGCATCTGTTCCCTTGGTCT |
| eNOS | TATTTGATGCTCGGGACTGC | AAGATTGCCTCGGTTTGTTG |
| ICAM-1 | GCCTGGGGTTGGAGACTAAC | CTGTCTTCCCCAATGTCGCT |
| GAPDH | ACCACAGTCCATGCCATCAC | TCCACCACCCTGTTGCTGTA |
